# Impact of spinach thylakoid extract-induced 12-week high-intensity functional training on specific adipokines in obese males

**DOI:** 10.1080/15502783.2024.2398467

**Published:** 2024-09-22

**Authors:** Ayoub Saeidi, Pezhman Motamedi, Maha Hoteit, Zahra Sadek, Wiam Ramadan, Marjan Mansouri Dara, Abdullah Almaqhawi, Shahnaz Shahrbanian, Hossein Abednatanzi, Kurt A Escobar, Zhaleh Pashaei, Maisa Hamed Al Kiyumi, Ismail Laher, Hassane Zouhal

**Affiliations:** a University of Kurdistan, Department of Physical Education and Sport Sciences, Faculty of Humanities and Social Sciences, Sanandaj, Kurdistan, Iran; b Kharazmi University, Department of Exercise Physiology, Faculty of Physical Education and Sports Science, Tehran, Iran; c National Council for Scientific Research-Lebanon (CNRS-L), Food Science Unit, Beirut, Lebanon; d Lebanese University, Faculty of Public Health, Section I, Beirut, Lebanon; e Lebanese University, Laboratory of Motor System, Handicap and Rehabilitation (MOHAR), Faculty of Public Health, Beirut, Lebanon; f Lebanese International University (LIU), Lebanese Institutes for Biomedical Research and Application (LIBRA), Beirut, Lebanon; g Islamic Azad University, Department of Physical Education and Sport Science, Science and Research Branch, Tehran, Iran; h King Faisal University, Department of Family Medicine and Community, College of Medicine, Al Ahsa, Saudi Arabia; i Tarbiat Modares University, Faculty of Humanities, Department of Sport Science, Tehran, Iran; j California State University, Department of Kinesiology, Long Beach, CA, USA; k University of Tabriz, Department of Exercise Physiology, Faculty of Physical Education and Sport Sciences, Tabriz, Iran; l Sultan Qaboos University, Department of Family Medicine and Public Health, Muscat, Oman; m Sultan Qaboos University Hospital, Department of Family Medicine and Public Health, Muscat, Oman; n The University of British Columbia, Department of Anesthesiology, Pharmacology and Therapeutics, Vancouver, Canada; o Univ Rennes, M2S (Laboratoire Mouvement Sport, Santé) - EA, Rennes, France; p Institut International des Sciences du Sport (2I2S), Irodouer, France

**Keywords:** Adipokines, functional training, insulin resistance, Obesity

## Abstract

**Background:**

Obesity presents multifarious etiopathologies with its management being a global challenge. This article presents the first ever report on the impact of spinach thylakoid extract-induced high-intensity functional training (HIFT) on obesity management via regulating the levels of novel adipokine, C1q/TNF-related Protein-12 (CTRP-12), furin, and Krüppel-like factor 15 (KLF-15).

**Methods:**

Sixty-eight obese male subjects were randomly divided into four groups: control group (CG), supplement group (SG), training group (TG), and the combined training and supplement group (TSG). After initial assessments of all groups, the training group commenced a twelve-week HIFT using the CrossFit program (comprising of three training sessions per week, each lasting 30 min). Eligible candidates were randomly assigned to either receive thylakoid-rich spinach extract (5 g per day) or a matching placebo (5 g per day of corn starch, 30 min before lunch) for a total duration of 12 weeks. All required data and investigations were collected at 48 h pre- and post-training.

**Results:**

The results indicated a substantial correlation between exercise and the time of KLF-15, furin, and CTRP-12 demonstrating effect sizes of 0.3, 0.7, and 0.6, respectively. Additionally, the training and supplementation group (TSG) exhibited a substantial decrease in low-density lipoprotein (LDL), total cholesterol (TC), and triglyceride (TG) levels (*p* < 0.0001). Concurrently, there was a significant increase in high-density lipoprotein-cholesterol (HDL-C) levels (*p* = 0.0001). Furthermore, a notable difference between the groups emerged in HDL, LDL, TC, and TG levels, supported by effect sizes of 0.73, 0.86, 0.96, and 0.89, respectively (*p* < 0.05).

**Conclusion:**

The study offered novel insights into the management of obesity using supplements induced by spinach-derived thylakoid extract during a 12-week HIFT program. The proposed combination intervention may reverse obesity-induced insulin resistance and metabolic dysfunctions by positive regulation of CTRP-12/adipolin and KLF15 and simultaneous suppression of furin levels.

## 1. Introduction

Obesity is a global public health concern and that contributes to numerous chronic diseases, including cancers, diabetes, metabolic syndrome, and cardiovascular diseases [1]. Obesity is an important component of the metabolic syndrome which leads to numerous pathological conditions, largely related to the release of adipokines (bioactive molecules secreted by adipose tissue) that have important roles in regulating metabolism, inflammation, and immunity [2,3]. However, a limited number of adipokines can also exert protective effects against obesity-related diseases by modulating inflammatory responses [4–6]. The adipose-derived insulin-sensitizing factor adipolin-related C1q/TNF-related Protein-12 (CTRP12) is such a newly described adipokine with anti-inflammatory and insulin-sensitive effects that can counteract complications involved with obesity, inflammation, as well as diabetes [7–9].

CTRP-12 mitigates inflammatory responses and promotes insulin sensitivity via insulin signaling pathways in adipose and hepatic tissues, thereby suppressing gluconeogenesis and increasing glucose uptake [10]. Additionally, it decreases the infiltration of macrophages and the expression of pro-inflammatory cytokines such as interleukin (IL)-1β, tumor necrosis factor (TNF)-α, and monocyte chemoattractant protein (MCP)-1 [11]. Expression of CTRP12 in adipocytes is regulated by various factors, among which the Kruppel-like factor (KLF-15) and furin have gained much attention and suggest that studies to explore the mechanism and treatment of obesity should consider in-depth interventions with beneficial adipokines along with their regulatory factors [12]. KLFs are primarily linked to diseases including inflammatory and metabolic disorders. Among the KLFs, KLF15 has a significant role in the differentiation of 3T3-L1 adipocytes and the stimulation of CTRP-12 activity [13]. Overexpression of KLF15 in adipocytes can reverse the inhibitory effects of TNF-α on CTRP-12 [13]. In contrast to KLF15, furin, promotes obesity-induced inflammatory conditions by stimulating TNFα release from adipose tissue, modulating the activities of adipocytokines (including adipolin), thereby intensifying the vicious cycle caused by the inflammatory response and insulin resistance [14].

Different exercise modalities have disparate effects on adipose tissue metabolism and adipokine secretion. High-intensity functional training (HIFT) modulates fat and glucose metabolism, and improves insulin sensitivity [15]. One of the mechanisms by which exercise reduces insulin resistance is by increasing the adipolin levels [16]. However, it is important to note that the type of exercise training used is an important factor in determining the benefits in limiting the harms of obesity and its related consequences [17]. For instance, CrossFit training, which is a combined exercise program with high intensity, parallel endurance, and strength performance, results in significant reductions in lipid oxidation in individuals with obesity [18–22]. Similarly, high-intensity interval training, which includes sets of high-intensity exercise with rest times between the sets [23], increases IL-6 and IL-10 activities [23], improves muscular endurance and cardiovascular fitness, and reduces body fat [24]. A study on the effect of a swimming training session on fat gene expression in white adipose in male mice reported that mRNA expression of PPARγ2 and C/EBPα was significantly reduced in epididymal white adipose tissue (eWAT), with an induction of KLFs such as KLF15 [25]. There is limited information on the benefits of different exercise regimens on the regulation of CTRP12 in reducing inflammation in people with obesity.

The benefits of effective and safe pharmacological agents such as the semaglutide for the management of obesity are in large part due to reducing body weight and a loss of fat mass [26–29]. HIFT is characterized by the use of continuously diversified movements, integrating various training methods of varying durations, with or without active rest intervals [30]. HIFT can be used in combination with various nutritional strategies to better manage obesity [31]. Examining the mechanistic interactions between HIFT and dietary supplements is crucial given the growing interest on the effectiveness of HIFT to increase mitochondrial capacity and improve exercise performance, as well as the well-known metabolic effects of diet and supplements in managing obesity-induced complications [31]. The first line of management in combating obesity is calorie-restricted diet [32]. Calorie-restrictions of 350–1000 kcal/day have significant positive effects on fat mass reduction, weight loss, and insulin sensitivity [33,34].

There is great interest in the use of phyto-nutraceuticals as an additional strategy for treating and preventing obesity-related complications [35]. Recent data indicate that the chloroplast membranes of leafy green plants, including spinach, contain thylakoids, which have anti-inflammatory and anti-obesity effects [36,37]. Despite the widely acknowledged advantages of physical training and natural supplements in ameliorating various metabolic and cardiovascular complications in individuals who are obese, there is sparse information on the efficacy of the combined impact of spinach thylakoid extracts and HIFT on obesity-induced health concerns. We hypothesized that the combination of spinach-derived thylakoid extract and a HIFT program can reverse obesity-induced insulin resistance and metabolic dysfunction. To test this hypothesis, we investigated the impact of spinach thylakoid extract and 12-week HIFT on obesity markers and the levels of novel adipokines such as CTRP-12, furin, and KLF15 that are involved in metabolic homeostasis.

## 2. Materials and methods

**Figure 1.**
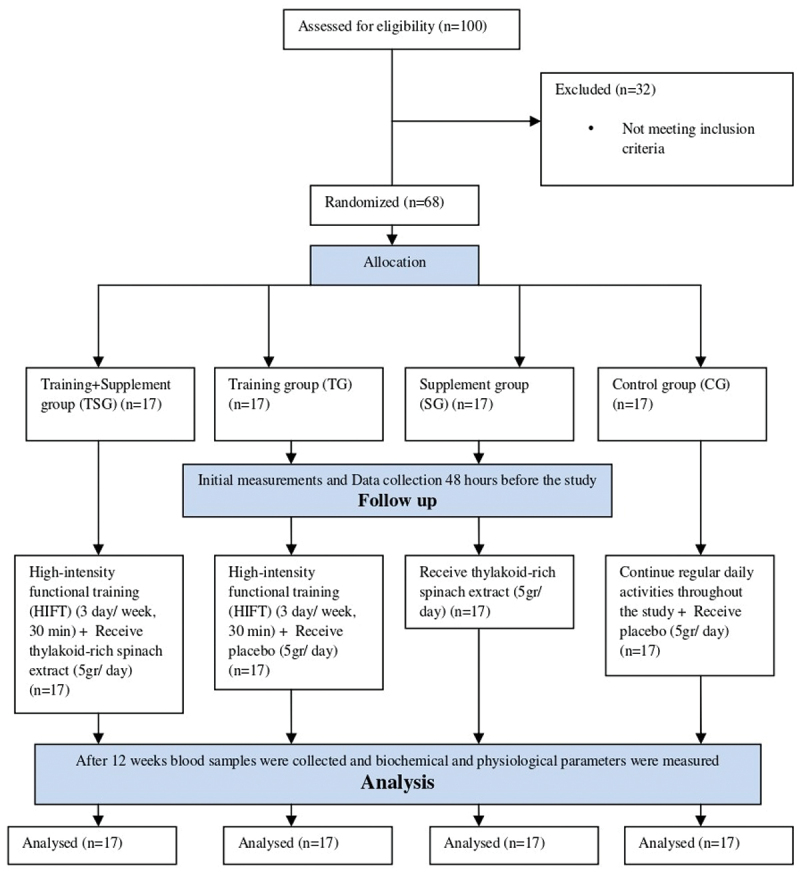
CONSORT flow diagram.

The study initially recruited 100 male volunteers from gymnasiums, medical clinics, hospitals, and social networks. Following an initial review, a total of 68 males were ultimately chosen for the current study, with a mean age of 27.6 ± 8.4 years, height of 168.4 ± 2.6 cm, weight of 95.7 ± 3.8 kg, and BMI of 32.6 ± 2.6 kg/m^2^. These participants were then distributed into four groups of 17 individuals per group (Figure 1). The inclusion criteria required individuals who met the following conditions: (a) BMI greater than 30 kg/m^2^, (b) absence of regular physical activities or exercise in the past six months, (c) no known medical conditions, including cardiovascular, endocrine, and metabolic diseases, and (d) non-consumption of alcohol. The exclusion criteria included participants with physical disabilities, joint diseases, and those taking supplements or medications that could potentially impact tissues such as muscle and fat. All participants provided written consent prior to participation, and they were directed to complete the Physical Activity Readiness Questionnaire (PAR-Q) [38]. During the initial visit, a comprehensive physical examination was conducted by a specialist. The study adhered to the principles outlined in the Declaration of Helsinki [39] and was approved by the Iranian Registry of Clinical Trials (IRCTID: IRCT20151228025732N77) and the Research and Ethics Committee of the Islamic Azad University, Damghan branch (Ethics code: IR.IAU.DAMGHAN.REC.1401.034) in December 2022.

### 2.1. Experimental design

Seventeen individuals were randomly assigned to each of four study groups: control group (CG), supplement group (SG), training group (TG), or combined training and supplement group (TSG) using an adaptive randomization method. Initial measurements, including height, weight, and some body composition parameters, were collected during an introductory session held one week before commencing the study. Additionally, other measurements of body composition and VO_2_peak were taken in the third session. All measurements occurred at the same time of day for all participants, within a one-hour window, and under consistent environmental conditions (approximately 20°C and 55% humidity). Data collection took place 48 h before the study and again after the final session. The TG and TSG groups started a 12-week training protocol (three sessions per week) and maintained a consistent diet 48 h before and after baseline and final assessments. Participants in the control and supplement groups continued with regular daily activities throughout the study.

### 2.2. Preparation of spinach thylakoids and placebo

The requisite spinach was procured in Tabriz, in the Iranian province of East Azerbaijan. The thylakoid supplement employed in this research was created in a laboratory of Islamic Azad University using the protocol outlined by Emerk et al. [40]. Fresh spinach leaves were washed and steeped in cold water after the stems and veins were removed. The homogenized spinach leaves (1000 g) were mixed with 1250 ml of water and then passed through four layers of 20 m Monodur polyester mesh. The acquired filtrate was then diluted ten times with distilled water before using hydrochloric acid to keep the solution at a pH of 4.7. Maximum precipitation occurs at pH 4.7, which is the isoelectric point of thylakoids. Thylakoids exhibited flocculation which led to a green precipitate after being exposed to subzero temperatures (−4°C). Repeated centrifugation was used to remove the supernatant from the filtrate thylakoids and collect the green precipitate at pH 4.7. Thereafter, this precipitate underwent one more round of precipitation at the same pH. After collecting the resulting washed thylakoids, the remaining sediments were subjected to freeze-drying to produce a powdered substance referred to as green thylakoids, which was then adjusted to a pH of 7.0. The placebo was made of maize starch that was edible and green in color with a kiwi fruit essence similar to that in the thylakoid powder. Corn starch is a white and odorless powder that is widely used in pharmaceutical sectors as and is an inert material with no therapeutic effects. Our alteration turned it into a green powder with a Kiwi aroma that resembled the properties of spinach thylakoid extracts. The diversity, clarity of modification, and adaptability of the materials were the driving forces for this alteration.

A green thylakoid powder was created with a Kiwi fruit flavor and identical shape, size, and color to be used as a placebo. Placebo powder was placed into identical sachets, each of which contained 5 g of either cornstarch powder or thylakoid. Participants consumed the sachet’s contents after dissolving in a glass of water 30-min prior to lunch.

A third party that was not involved in any other aspect of the study coded and delivered the items every month. There was a supplement consumption chart available to serve as a reminder to participants to take their supplements. To ensure compliance, this chart had to be returned at each visit. Participants received a weekly phone call and a daily text message reminder to consume the supplement, making it easier to maintain study protocol adherence and reduce study dropout. The leftover sachets were requested from the participants at each visit, which allowed us to gauge compliance. When a subject ingested 80% or more of the supplements, they were deemed to be adherent [37]. Participants were consumed 5 g of spinach thylakoid extract per day or one sachet of raw corn starch per day (in the control group) 30 min before lunch for 12 weeks.

### 2.3. Training protocols

The HIFT program used in our study consisted of 6 sessions, with a duration of up to 60 min per session. The initial two sessions supervised by a trainer having a Level 1 CrossFit certificate served as an introduction to fundamental movements in HIFT, encompassing exercises such as the air squat, overhead squat, front squat, press, push jerk, push press, sumo dead lift high pull, deadlift, and medicine ball clean. Notably, no additional workouts were performed on days 1 and 2. Each HIFT class, starting on day 3, included 10–15 min of stretches and warm-ups, 10–20 min of instruction and technique practice, and 5–30 min of the workout of the day (WOD), which was conducted at a severe intensity, depending on the participants’ abilities and fitness levels. Exercise methods encompassed a variety of modalities, incorporating aerobic activities such as running, body-weight and weightlifting exercises.

The exercises were systematically varied utilizing the CrossFit training model [41], structured in single, double, or triple modalities, and executed with consideration to time, repetitions, or weight. Each HIFT participant was individually prescribed specific weights and exercises, with comprehensive documentation of their unique regimen [42]. Each participant’s time to complete the WOD, rounds, and repetitions, weights utilized, and any alterations from the planned workout that were necessary were also recorded, depending on the WOD format.

### 2.4. Assessments of body composition and cardiorespiratory fitness

A calibrated scale and stadiometer (Seca, Germany) was used to measure body mass and height in order to compute BMI. The amount of fat mass (FM) and fat-free mass (FFM) was measured using a bio-impedance analyzer (Medigate Company Inc., Dan-dong Gunpo, Korea). An electronic sphygmomanometer (Kenz BPM AM 300P CE, Japan) was used to measure blood pressure, and a Polar V800 heart monitor (Finland) was used to record the heart rate. A calibrated gas analyzer (Metalyzer 3B analyzer, Cortex: biophysics, GMbH, Germany) was used to do a gas analysis before every test. Determinations of VO_2peak_ wee made in accordance with the standards established by the American College of Sports Medicine (ACSM) [43,44] using a modified Bruce protocol on a treadmill (H/P/Cosmos, Pulsar med 3p-Sports & Medical, Nussdorf-Traunstein, Germany) in accordance with earlier research involving overweight and obese populations. Physiological assessment of VO_2peak_ was based on participants indicating their maximum effort and reaching exhaustion, as measured by the Borg scale, or if the examinator saw significant dyspnea with a respiratory exchange ratio (RER) of less than 1.10 and a plateau in oxygen consumption (VO_2_) using guidelines for cardiopulmonary exercise testing (CPET) [45,46]

### 2.5. Nutrient intake and dietary analysis

A 3-day dietary recall, consisting of one weekend day and two weekdays, was used to evaluate dietary practices prior to and after the trial [47]. Diet Analysis Plus version 10 (Cengage, Boston, MA, USA) was used to analyze dietary items and determine the overall energy consumption from each item.

### 2.6. Blood markers

Blood samples were obtained during normal operating hours, between 8 and 10 am, when blood samples were drawn from the right arm following a 12 h fast for each test. There was a 72-h period both before and following the first workout. Samples of blood were put in tubes, spun at 3000 rpm for 10 min, and then kept at −70°C. An ELISA kit with Catalogue No. SK00392–06 from Aviscera Bioscience, USA, was used to measure the levels of CTRP-12. Assay specifications included a sensitivity of 10 pg/mL, an intra-assay CV of 6%, and an inter-assay CV of 8%.

The levels of plasma furin were measured using an ELISA kit (Catalogue No. E2321Hu) from Bioassay Technology Laboratory, China, which has a sensitivity of 6.93 ng/L, an intra-assay CV of less than 8%, and an inter-assay CV of less than 10%. An ELISA kit (Bioassay Technology Laboratory, China, Catalogue No. E4560Hu) was used to quantify plasma KLF-15. The kit had a sensitivity of 0.11 ng/mL, an intra-assay CV of less than 8%, and an inter-assay CV of less than 10%.

A photometric approach (Pars Testee’s Quantitative Detection Kit, Tehran, Iran) was used to quantify high-density cholesterol (HDLC) with coefficients and sensitivities of 1.8% and 1 mg/dl, respectively, and low-density cholesterol (LDL-C) with coefficients and sensitivities of 1.2% and 1 mg/dl, respectively. Enzymatic techniques (CHOD-PAP) were used to measure plasma levels of triglycerides (TG) and total cholesterol (TC).

### 2.7. Statistical analysis

Version 24 of SPSS software was used for statistical analysis, and a p-value of less than 0.05 was considered statistically significant. Determining a statistical difference with a 95% confidence interval (CI) and an 80% power value served as the basis for calculating sample size. The Shapiro–Wilk test and two-way ANOVA repeated measures were employed to evaluate the data’s normality and the group-time interactions, respectively. Baseline data from the four groups were assessed using Fisher LSD post-hoc tests and one-way ANOVA testing. Pairwise comparisons were used to determine mean differences when ANOVA testing revealed significant differences. Partial eta-squared was used to express effect sizes (ES) and were classified into four categories: trivial (<0.2), small (0.2–0.6), moderate (0.6–1.2), large (1.2–2.0), and very large (2.0–4.0) (Hopkins et al., 2009).

## 3. Results

### 3.1. KLF-15, Furin, and CTRP-12

Baseline levels of KLF-15, furin, and CTRP-12 were similar in the four study groups (*p* > 0.05). The group-time interactions for KLF-15 (*p* = 0.001, ES: 0.3), furin (*p* = 0.005, ES: 0.7), and CTRP-12 (*p* = 0.005, ES: 0.7) were all statistically significant (Table 2). Time-related effects showed significant differences between the pretest and posttest values for KLF-15 (*p* = 0.005, ES: 0.5), furin (*p* = 0.005, ES: 0.7), and CTRP-12 (*p* = 0.005, ES: 0.8). In addition, there was a statistically significant difference in the study groups for KLF-15 (*p* = 0.002, ES: 0.3), Furin (*p* = 0.005, ES: 0.7), and CTRP-12 (*p* = 0.005, ES: 0.7) after attending the intervention program.

Post-hoc multiple comparison analysis further indicated that all pairwise comparisons of the main outcomes were statistically significant (*p* < 0.05) in the study groups except for TSG and TG related to KLF-15 (*p* = 0.2), furin (*p* = 0.5) and CTRP-12 (*p* = 0.2), and SG and TG for KLF-15 (*p* = 0.07) and CTRP-12 (*p* = 0.29). Furthermore, CTRP-12 levels did not show a statistically significant difference between the pairwise comparisons of SG and TSG (*p* = 0.07). The only non-significant difference between SG and the control group was observed for KLF-15 (*p* = 0.08).

### 3.2. Physiological metrics analysis

Measurements of body composition and cardio-respiratory fitness variables revealed no significant differences in BMI or VO_2_ peak between the groups (*p* = 0.11, ES = 0.14) or (*p* = 0.27, ES = 0.09) respectively. However, significant differences were noted in other variables such as body weight with an effect size of 0.37, FFM with an effect size of 0.23, fat mass with an effect size of 0.42, HDL-C with an effect size of 0.37, LDL-C with an effect size of 0.34, TC with an effect size of 0.46, and TGs with an effect size of 0.66 (*p* < 0.05) (Table 3).

There were significant posttest differences for SG, TG, and TSG (*p* < 0.05) compared to their respective pretest values in the intergroup comparison of anthropometry, body composition, and cardiorespiratory fitness measures, with changes in the CG (*p* > 0.05). Significant group and time interactions occurred for all variables (*p* < 0.05, ES range: 0.43–0.96). Post hoc multiple comparisons showed that all intervention groups varied substantially from the CG (*p* < 0.05) with the exception of BMI (*p* = 0.62), VO_2_ peak (*p* = 0.53), TGs (*p* = 0.78), and HDL (*p* = 0.22) in the SG. Furthermore, all pairwise comparisons involving SG and TG were statistically significant (*p* < 0.05), with the exception of those involving body weight (*p* = 0.06), BMI (*p* = 0.87), and FFM (*p* = 0.78). There were no differences in body weight (*p* = 0.57), VO_2_ peak (*p* = 0.78), fat mass (*p* = 0.64), HDL (*p* = 0.78), LDL (*p* = 0.68), TC (*p* = 0.31), and TGs (*p* = 0.1) when TG and TSG were compared. All variables were different (*p* < 0.05) between participants in the TSG and SG groups, with the exception of FFM (*p* = 0.14).

## 4. Discussion

We report novel data on the combined effects of HIFT and spinach-thylakoid extract on specific adipokines in males with obesity in an intervention study involving measurements of cardiorespiratory parameters, daily nutrition intake, lipid profile, and the plasma levels of a novel anti-inflammatory adipokine, CTRP12 and its key regulatory factors KLF15 and furin.

Baseline plasma levels of KLF-15, furin, and CTRP-12 in all the groups were similar, but the levels of these proteins underwent significant changes in all the intervention groups, with a significant reduction in furin levels and increases in CTRP12 and KLF15 levels. This suggests that the spinach-thylakoid extract and HIFT, alone or in combination increases KLF15 and CTRP12 levels in obese individuals, while decreases in furin mediate reversal of obesity-induced inflammatory conditions (Table 1). In addition, there were decreases in LDL, TC, TG levels, weight, BMI, FFM, and body fat and increases in HDL-C levels of the TG and TSG groups. There were no significant differences between TG and TSG in physiological and biochemical parameters. Changes in anthropometric parameters such as BMI were not significant in SG compared to CG, TG, and TSG (Table 3). There are no other studies on the concomitant impact of spinach-thylakoid extract and HIFT on the levels of these adipose-tissue derived mediators, although some reports on the effect of exercise on the levels of adipokines agree with our findings, such as a report of increases in the serum adipolin levels after eight weeks of continuous low-intensity exercise [16]. The intensity and duration of exercise strongly impacts glucose and lipid metabolism, adipokines levels, and the insulin response to exercise, as shown in obese male subjects with fatty-liver disease, where 12 weeks of HIIT reduced levels of leptin, TG, LDL, and insulin resistance while also increasing adiponectin levels [48]. Adiponectin has an important role in regulating insulin resistance and combating obesity-associated dysfunctions [49]. Another study using 12 weeks of resistance training on sedentary postmenopausal obese women reported significant improvement in their insulin sensitivity, levels of adipolin, fasting glucose, HDL, and anthropometric indices such as BMI [50].

**Table 1.** Nutritional intakes in the study groups.

	CG		SG		TG		TSG	
	Pre	Post	Pre	Post	Pre	Post	Pre	Post
Energy (kcal/d)	2,255±67	2,261±86	2,274±111	2,132±150	2,253±127	2,161±167	2,271±177	2,110±186
CHO (g/d)	280±12.4	282±19.3	276.4±77.1	260±67.5	283±48.6	264±19.2	288±18.6	261±19.1
Fat (g/d)	81.2±10.0	80±8.8	85.5±11.7	74±13.2	80.4±14.4	72.1±13.2	79.8±10.87	70.2±15.3
Protein (g/d)	103±11.0	105±13.3	100±14.5	94±11.6	102±17.8	92±12.7	103±15.5	89±14.5

Abbreviations: CHO: carbohydrates; CG: control group; SG: supplement group; TG: training group; TSG: training and supplement group.

Data are presented as mean (±SD); CG: Control group; SG: Supplement group; TG: Training group; TSG: Training supplement group. *Indicates significant differences compared to the Pre-values (*p* < 0.05).

**Figure 2.**
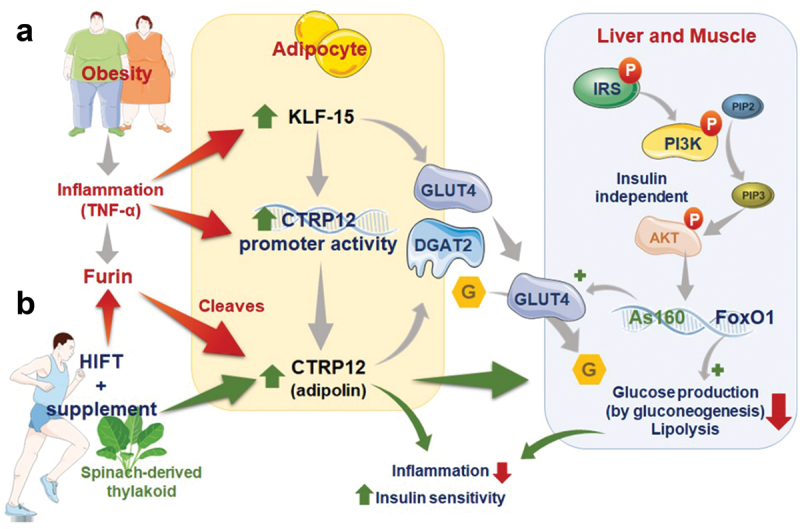
Mechanism of obesity management through regulation of adipolin and related factors by the HIFT + spinach-thylakoid extract.

Our study and data from the published literature suggest that CTRP12/adipolin can activate the insulin-independent pathways to increase glucose uptake into adipocytes. Thus, by reducing glucose production and promoting glucose utilization, CTRP12 may be an important player in reversing metabolic disorders such as obesity and insulin-resistance (Figure 2).

The novel anti-inflammatory adipokine CTRP-12/adipolin stimulates the insulin-independent phosphoinositide 3-kinase (PI3K) pathway to activate the transcription factors nuclear Forkhead boxO1 (FOXO1) and Akt substrate of 160 (As160) to upregulate the expression of GLUTs and increase glucose uptake by hepatocytes and muscles, while also inhibiting enzymes of gluconeogenesis and lipolysis. Krüppel-like factor 15 (KLF15) positively regulates CTRP12 while also directly stimulating glucose uptake. The mechanism by which the novel combination of spinach thylakoid extract and HIFT could improve lipid profile and metabolic status in obese males may include the upregulation of adipolin, KLF15, and inhibition of furin, thereby restoring metabolic homeostasis. Several explanations have been suggested for the positive effects of spinach-derived thylakoids in the management of obesity. For example, thylakoid membranes isolated from spinach inhibits the activity of pancreatic lipase/co-lipase (up to 80%) *in vitro*. Thus, thylakoids inhibit the digestion and absorption of dietary fat. The benefits of this supplement are likely to be augmented by other treatments such as physical activity.

Our study highlights CTRP12 as a novel insulin-sensitizing adipokine, metabolic regulator, and anti-inflammatory mediator released from adipose tissues that mediates metabolic homeostasis of the body [13,51,52]. Circulating levels of CTRP12 as well as its mRNA expressions decrease in obesity [53], in addition to increases in levels of inflammatory markers such as TNF-α in adipocytes [54]. TNF-α inhibits the transcription factor KLF15 by activating JNK, which in turn reduces CTRP-12 levels in adipocytes [51]. Therefore, it is conceivable that reduced adipolin expression in obese state involves inflammation of adipose tissues. Collectively, these results suggest that CTRP12 ameliorates lipid accumulation by promoting cholesterol efflux from macrophages and alleviates inflammatory response.

The findings of our study indicate that levels of furin in the intervention groups were significantly lower than in the control group. Furin is a member of the proprotein convertase family that proteolytically cleaves adopolin [14,55]. Hence, upregulation of furin expression in obesity reduces circulating levels of the full-isoform of adipolin and increases levels of its proteolytically cleaved isoforms. Since the full-isoform of adipolin mediates insulin-sensitization and glucose uptake in the adipocytes [55], obesity-induced increased furin levels reduce insulin sensitivity and glucose uptake. However, it may be suggested that HIFT combined with a spinach-thylakoid extract reduces furin levels in obese men to restore circulating levels of the full-isoform of adipolin to improve metabolic homeostasis, as shown by post-intervention improvements in cardio-respiratory parameters and lipid profiles (Tables 2 and 3).

**Table 2.** Pre- and post-training values for KLF-15, furin, and CTRP-12 in the study groups. Data are presented as mean (SD).

Variables	Group	Pre-training	Post-training	P values (η2)		
				Time	Group × Time Interaction	Group
KLF-15 (ng/ml)	CG	12.2 (1.6)	12.4 (1.7)	0.001 (0.5)	0.001 (0.3)	0.002 (0.4)
	SG	12.1 (1.2)	13.8 (1.6)			
	TG	12.4 (1.2)	15.2 (1.9)			
	TSG	11.6 (1.4)	16.2 (1.9)			
Furin (ng/l)	CG	556.6 (13.9)	578.3 (17)	0.001 (0.8)	0.001 (0.7)	0.001 (0.6)
	SG	550.5 (18.7)	513.3 (23.3)			
	TG	547.2 (27.0)	480.5 (19.5)			
	TSG	546.3 (21.1)	475.3 (21.3)			
CTRP-12 (ng/ml)	CG	4.2 (0.4)	3.8 (0.4)	0.001 (0.6)	0.001 (0.6)	0.001 (0.8)
	SG	3.9 (0.7)	5.9 (0.2)			
	TG	3.7 (0.5)	6.1 (0.5)			
	TSG	3.8 (0.6)	6.4 (0.5)			

Abbreviations: CG: control group; SG: supplement group; TG: training group; TSG: training and supplement group.

**Table 3.** Physiological and biochemical parameters of respondents.

Variables	Group	Pre-training Mean (SD)	Post-training Mean (SD)	p-values (η2)		
				Time	Group × Time Interaction	Group
Weight (kg)	CG	94.33(1.82)	93.55 (2.43)	0.001 (0.63)	0.001 (0.44)	0.001 (0.37)
	SG	93.28(2.61)	91.13 (2.12)			
	TG	92.78(1.89)	89.19 (2.37)			
	TSG	94.13(1.90)	87.25 (2.30)			
BMI (kg/m^2^)	CG	33.08(1.34)	32.87 (1.44)	0.001 (0.63)	0.001 (0.45)	0.11 (0.14)
	SG	32.66(1.37)	31.93 (.93)			
	TG	33.22(1.07)	31.85 (1.19)			
	TSG	33.05(.75)	30.68 (.95)			
VO_2_peak (mL⋅kg^−1^⋅min^−1^)	CG	27.27(2.41)	26.90 (2.25)	0.001 (0.62)	0.001 (0.62)	0.27 (0.09)
	SG	27.09(2.84)	27.54 (2.91)			
	TG	27.36(2.37)	29.90 (1.92)			
	TSG	27.00(2.04)	30.18 (2.22)			
FFM (kg)	CG	27.63(1.20)	26.54 (2.25)	0.001 (0.46)	0.001 (0.43)	0.01 (0.23)
	SG	27.09(1.81)	29.36 (.92)			
	TG	26.72(1.27)	29.54 (1.5)			
	TSG	27.18(1.77)	30.36 (1.2)			
Body fat (%)	CG	30.09 (1.51)	30.83 (2.05)	0.001 (0.54)	0.001 (0.46)	0.001 (0.42)
	SG	30.10 (1.59)	28.08 (.79)			
	TG	30.36 (1.50)	26.89 (.95)			
	TSG	31.13 (1.35)	26.62 (1.21)			
HDL (mg/dl)	CG	39.34 (1.22)	38.43(1.30)	0.001 (0.72)	0.001 (0.73)	0.001 (0.37)
	SG	38.88 (1.23)	39.74 (3.99)			
	TG	38.66 (1.67)	44.51 (1.34)			
	TSG	38.56 (1.41)	44.80 (2.12)			
LDL (mg/dl)	CG	125.22 (4.47)	125.02 (4.70)	0.001 (0.91)	0.001 (0.86)	0.001 (0.34)
	SG	125.56 (5.42)	121.41 (5.24)			
	TG	126.75 (4.38)	111.20 (2.92)			
	TSG	127.14 (3.64)	110.50 (2.52)			
TC (mg/dl)	CG	226.70 (5.27)	226.81 (5.26)	0.001 (0.97)	0.001 (0.96)	0.001 (0.46)
	SG	227.44 (5.48)	222.09 (5.19)			
	TG	227.81 (5.29)	207.44 (4.95)			
	TSG	227.38 (5.49)	205.23 (4.77)			
TGs (mg/dl)	CG	242.10 (4.39)	242.81(3.85)	0.001 (0.90)	0.001 (0.89)	0.001 (0.62)
	SG	245.83 (5.93)	242.05 (5.40)			
	TG	244.58 (7.48)	217.39 (9.88)			
	TSG	242.92 (5.96)	212.85 (4.64)			

Data are presented as mean (SD); BMI: Body mass index, FFM: Fat-free mass, TG: Triglycerides, TC: Total cholesterol, LDL: Low-density lipoprotein, HDL: High-density lipoprotein. CG: control group; SG: supplement group; TG: training group; TGs: triglycerides; TSG: training and supplement group; VO_2_peak: peak oxygen uptake.

### 4.1. Study limitations

Our study has some limitations: (A) Plasma levels of furin, adipoline, KLF15, and physiological anthropometric parameters were similar in pairwise comparisons of the TSG and TG groups, and levels of KLF15 and adipoline and BMI and FFM were not different in SG and TG groups. This may be partly explained by the analysis of routine dietary intakes before and after the study, where even without the supplement, subjects augmented energy consumed from proteins, lipids, and carbohydrates in the groups that obtained only spinach-thylakoid extracts (SG), only HIFT (TG), and in the group that received the supplementation in combination with HIFT. (B) Our study enrolled only male participants. (C) We did not explore the details of the interactions of regular dietary components and thylakoids, and how spinach-derived thylakoids can be optimized for use in managing obesity.

## 5. Conclusion

Our study offers novel insights into the advantages of a non-pharmacological intervention for the treatment of obesity, utilizing a combination of spinach-thylakoid extract and a 12-week HIFT program. The combined intervention protocol used in our study was able to reverse obesity-induced insulin resistance and metabolic dysfunction by increasing levels of the anti-inflammatory adipokine CTRP 12/adipolin and its regulatory factor KLF15, while at the same time suppressing furin levels to prevent cleavage of the insulin-sensitizing full form of adipolin.
